# Shenqi Fuzheng Injection Alleviates the Transient Worsening Caused by Steroids Pulse Therapy in Treating Myasthenia Gravis

**DOI:** 10.1155/2013/816829

**Published:** 2013-11-20

**Authors:** Guo-Yan Qi, Peng Liu, Bu-Lang Gao

**Affiliations:** Department of Oncology, Shijiazhuang First Hospital and Hebei Provincial Hospital of Myasthenia Gravis, 36 Fanxi Road, Shijiazhuang, Hebei 050011, China

## Abstract

*Purpose*. To evaluate the treatment effect and side effect of Shenqi Fuzheng Injection (SFI) on alleviating transient worsening of myasthenia gravis (MG) symptoms caused by high-dose steroids pulse therapy. *Methods*. Sixty-six consecutive patients with MG were randomly divided into two groups: the treatment group treated with SFI and methylprednisolone pulse therapy (MPT) and the control group treated with MPT alone. The severity of MG before, during, and after MPT and the duration of transient worsening (TW) were evaluated and compared with the clinical absolute scoring (AS) and relative scoring (RS) system. *Results*. Twenty-nine patients experienced TW in each group. At TW, the AS was significantly increased (*P* < 0.000) in both groups compared with baseline data, with the AS increase in the treatment group (16.8 ± 2) significantly smaller (*P* < 0.05) than in the control group (24.9 ± 2.5). At the end of the treatment course, the AS for the treatment group was significantly decreased (7.5 ± 0.9) compared with at TW, although no significant difference compared with the control (9.7 ± 1.1). The TW lasted 1–6 days (mean 3.7) for the treatment group, significantly shorter (*P* < 0.05) than 2–12 days (mean 7.8) for the control. The RS for the treatment group at the end of treatment was 43.8%–100% (mean 76.8% ± 2.6%), significantly better than the control group: 33.3%–100% (mean 67.2 ± 3.6%). Slight side effects (18.75%) included maldigestion and rash in the treatment group. *Conclusion*. SFI has a better treatment effect and few side effects and can alleviate the severity and shorten the duration of the transient worsening of MG during steroids pulse therapy.

## 1. Introduction

Myasthenia gravis (MG), characterized by fatigability and fluctuating weakness of the skeletal muscles, was one of the neurological diseases with a serious prognosis in the past. In the present, the prognosis of MG has improved dramatically due to advances in critical care and symptomatic treatment [1]. MG is a B-cell-mediated, T-cell-dependent autoimmune disorder resulting from loss of tolerance toward self-antigens in the thymus. In this disease, the major autoantigen is the nicotinic acetylcholine receptor (AChR) at the neuromuscular junction, and the impaired neuromuscular transmission is caused by IgG autoantibodies binding to the AChR on the postsynaptic membrane of skeletal muscle in more than 80% of patients, thus causing clinical symptoms of muscle weakness and fatigability. Antibodies and complements are the key effectors of the loss of postsynaptic AChRs and associated destruction of the neuromuscular junction [2, 3]. Thus, the goal of MG treatment is to interrupt the autoimmune process by T cells and B cells as soon as possible and thereby prevent further destruction of the neuromuscular junction. Corticosteroids are especially useful as a short-term immunosuppressive agent and have a fairly rapid onset of action which has been well proven and safe [4]. However, long-term side effects existed, with the risk of osteoporosis being effectively reduced by administration of bisphosphonate. Corticosteroids are primarily used early during MG and together with other immunosuppressive agents. Marked improvement has been reported in 70%–80% of MG patients in observational studies. However, high-dose corticosteroids (pulse steroid therapy) can cause a brief, temporary MG worsening, and this is why this therapy was originally rejected for MG in the 1950s [5–7]. To counteract this deterioration and, more importantly, to minimize side effects, alternate-day therapy with corticosteroids is always recommended in MG. Except for acute situations, therapy should be started in a low dose and then gradually increased. Starting with prednisolone 10–20 mg on alternate days as well as increasing the dose to 60–80 mg every other day during 4–6 weeks is usually safe. 

Shenqi Fuzheng Injection is made from Radix Astragali (Huangqi) and Radix Codonopsis (Dangshen), two Chinese medicinal herbs which have been used in China and some other Asian countries as herbal medicines for centuries [8]. Radix Astragali is usually used as an immunomodulating agent in the treatment of immunodeficiency diseases and to alleviate the adverse effects of chemotherapeutic drugs [9, 10]. Radix Codonopsis is usually used for treating dyspepsia, fatigue, bronchitis, cough, inflammationn and so on, with its pharmacological activities including antifatigue and immunomodulatory activities being reported in the Chinese literature [11]. The purpose of this study was to investigate the role of Shenqi Fuzheng Injection in alleviating the short-term worsening of MG during the pulse therapy of steroids. 

## 2. Materials and Methods

### 2.1. Subjects

Sixty-six consecutive MG patients treated in our hospital between December 2002 and June 2005 were enrolled in this study which was approved by the hospital scientific committee for patient care and research. All patients provided their signed informed consent and were hospitalized for their treatment. 

### 2.2. Inclusion and Exclusion Criteria

Patients with definite factors that would aggravate the clinical symptoms of MG, such as infection or self-discontinuation of medication, were excluded because it would be difficult to determine whether a worsening of MG symptoms was caused by the effect of steroids or was a reflection of the natural course of the disease. Patients with severe pretreatment fluctuation of MG symptoms were also excluded. Moreover, patients who had recently undergone nonpharmacological therapeutic interventions that might influence the natural course of MG, for example, thymectomy or plasma exchange, were also excluded. All these patients were diagnosed to have MG based on typical clinical symptoms and two of the following diagnostic standards for MG: (1) positive neostigmine test, (2) positive acetylcholine receptor antibody, and (3) abnormal neurophysiological findings (repetitive nerve simulation and single-fiber electromyography). 

### 2.3. Demographic Data and Randomization

The patients were randomly divided into two groups: the treatment and the control. The stratified and block randomization design was used to divide these patients. This was not a blind but an open-labeled study. There was no significant difference (*P* > 0.05) in the age, gender, course of disease, and pretreatment classification by the MG Foundation of American Clinical Classification (Table 1).

### 2.4. Treatment

Both the treatment and control groups had methylprednisolone pulse therapy (15–20 mg/kg) which was gradually tapered and oral administration of pyridostigmine bromide (an acetylcholinesterase inhibitor). If patients were already taking methylprednisolone or similar drugs, the drugs would be withheld for 48 hours before this treatment. The treatment group also had 250 mL Shenqi Fuzheng Injection intravenously once daily, with the treatment course lasting for 20 days for both groups. The clinical absolute and relative scoring system was used to evaluate the treatment effect during the steroids pulse therapy (Table 2) [12]. The clinical absolute score system evaluates the patients in the following eight aspects: ptosis, upper eyelid fatigue, eyeball horizontal movement, upper limbs fatigue, lower limb fatigue, facial muscles, chewing and swallowing functions, and respiratory muscle function [12]. The clinical absolute scores ranges 0–64, with 0 being the least severe (or normal) while 64 is being the most severe. The clinical relative score is equal to (pretreatment clinical absolute score − posttreatment clinical absolute score)/pretreatment clinical absolute score. The clinical relative score ranges from 0 to 100%, with 0 indicating no improvement while 100% indicates clinical recovery of the disease (not necessarily cure of the disease). The clinical absolute score reflects the severity of muscle weakness while the clinical relative score reflects the change of muscle weakness after treatment and is better in assessing the treatment effect. The severity and duration of the transient worsening of disease were recorded from emergence to disappearance of the worsening effect and were compared between the two groups at the end of the treatment course.

### 2.5. Primary Outcome Measure

The primary outcome measure was the clinical effect of Shenqi Fuzheng Injection in alleviating the transient worsening induced by steroids. The second primary outcome measure was the possible side effects of the Shenqi Fuzheng Injection.

### 2.6. Statistical Analysis

 Measurement data were presented as mean ± SD (standard deviation). All statistical analyses were undertaken by using SAS V8 software to compare the difference in the duration of transient worsening between the two groups by the use of *t*-test or Wilcoxon signed-rank test. The *P* value was set at 0.05 as significant.

## 3. Results

### 3.1. Evaluation Standards for the Treatment Effect

The transient worsening of disease began when the clinical absolute score during the steroid pulse therapy increased three or more points than that before the treatment was started. When the clinical absolute score decreased three or more points than that before treatment, the transient worsening was ended. The duration of the transient worsening was thus determined and compared between the treatment and control groups.

### 3.2. Clinical Treatment Effect

**Figure 1:**
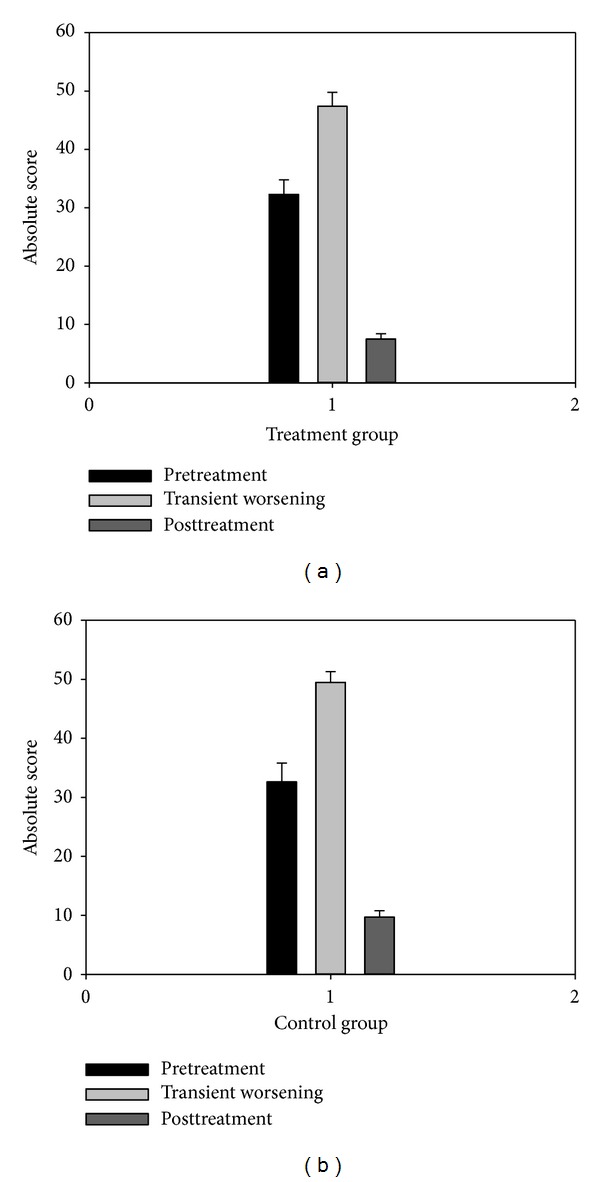
Change of the clinical absolute scores before, during, and after transient worsening of myasthenia gravis symptoms. A significant difference (*P* < 0.000) exists between the pre- or posttreatment data and the data during the transient worsening in both the treatment (a) and control (b) groups.

**Figure 2:**
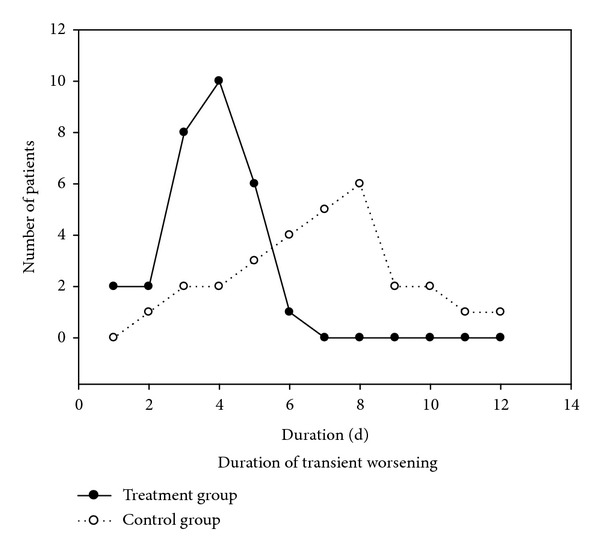
The effect results were shown in both groups with different treatment. RE: recovery; BR: basic recovery; MI: marked improvement; IM: improvement; IN: ineffectiveness.

**Figure 3:**
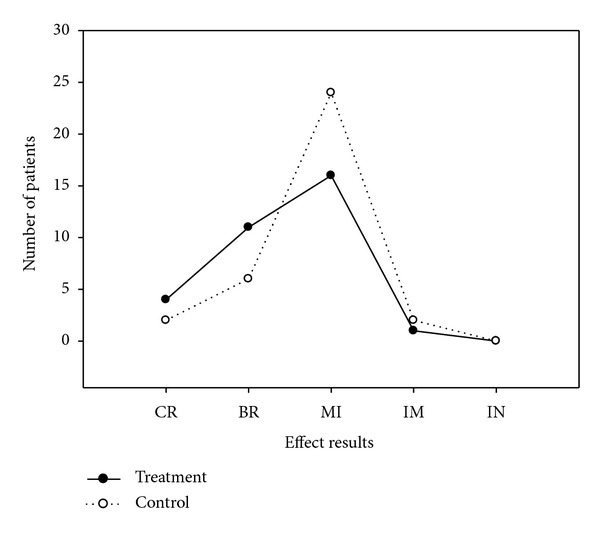
Duration of transient worsening during steroids pulse therapy. Most of the worsening occurs between 3 and 5 days in the treatment group and between 3 and 10 days in the control group. D: day.

At baseline (before the steroid pulse therapy), the clinical absolute score had no significant difference (*P* > 0.05) between the treatment (32.3 ± 2.5) and the control (32.6 ± 3.2) groups (Figure 1). During steroid pulse therapy, except 3 and 5 patients in the treatment and control groups, respectively, who did not experience transient worsening, the remaining 29 patients (90.6% in the treatment group and 85.3% in the control group) had transient worsening caused by steroid therapy. At transient worsening (Figure 1), the clinical absolute score was significantly increased (*P* < 0.000) compared with baseline data (47.3 ± 2.6 for the treatment and 50.5 ± 2.2 for the control), although no significant difference (*P* > 0.05) between the two groups. However, the increase of the absolute score for the treatment group (3–40, mean 16.8 ± 2) was significantly (*P* < 0.05) smaller than for the control group (6–46, mean 24.9 ± 2.5). At the end of the treatment course, the clinical absolute score for the treatment group was significantly (*P* < 0.000) decreased (7.5 ± 0.9) compared with transient worsening (Figure 1), although no significant difference (*P* > 0.05) compared with the control group (9.7 ± 1.1). The transient worsening of MG lasted from 1 to 6 days (mean 3.7) for the treatment group (Figure 2), with most of the patients (24) experiencing transient worsening between 3 and 5 days, significantly shorter (*P* < 0.01) than that for the control group (2–12 days, mean 7.8, mostly between 3 and 10 days). The clinical relative score for the treatment group at the end of treatment course ranged from 43.8% to 100% (mean 76.8% ± 2.6%), significantly better (*P* < 0.05) than for the control group (33.3%–100%, mean 67.2% ± 3.6%) (Table 3). More patients got complete remission and basic remission in the treatment group (15) than in the control group (8) (Table 4 and Figure 3).

### 3.3. Possible Side Effects of Shenqi Fuzheng Injection

Five patients in the treatment group had slight maldigestion without additional medications for it. One patient had temporary rash on the neck lasting 3 days with no special treatment. Thus, the rate of slight side effect for this injection was 18.75%. No similar reaction was found in the control group.

## 4. Discussion

This study investigated the role of Shenqi Fuzheng Injection in alleviating the transient worsening of MG caused by high-dose steroids pulse therapy and demonstrated that Shenqi Fuzheng Injection can alleviate the severity and shorten the duration of the transient MG worsening during steroids pulse therapy. Moreover, Shenqi Fuzheng Injection also has a good treatment effect on MG in the treatment group compared with the control group treated with steroids alone. 

Traditional Chinese Medicine (TCM) is gaining increasing attention in both commercial and academic circles because it has been identified for several decades that TCM herbs may play a role in reducing the risk of chronic disease, act as powerful antioxidants, and have a pharmacological impact on the immune system [13]. In the past, much research has been focused on the immunomodulatory effect of TCM [13]. Evidence has been accumulating that some TCM herbs affect immune cells and cytokine production associated with immune responses [14, 15], with some TCM herbs modulating the activity of the innate immune system and others targeting cell subsets of adaptive immunity. Immunomodulation maintains homeostasis of the immune system and is essential for normal health. Abnormalities in immunomodulation can induce serious diseases. When normal immune surveillance is suppressed, the immune system will fail to protect the body from infectious agents or other harmful substances, resulting in generalized susceptibility to infection and tumorigenesis [16, 17]. However, when the immune system is excessively activated, it will respond to antigens not associated with infectious agents and will break down self-tolerance, leading to autoimmune disease, hypersensitivity, and graft failure [18–20]. MG is such a disease caused by autoantibodies against the autoantigens of the body including the acetylcholine receptors in the postsynaptic muscle endplate membrane and a distinct endplate membrane intrinsic protein, muscle-specific receptor tyrosine kinase. Immunosuppressive treatment in MG is common and some patients have consequently gained long-term remission of disease. Corticosteroids are currently the mainstay of the immune-directed treatment for patients with MG. Although the corticosteroids appear to be effective in inducing a remission, they have major side effects, making the cost-to-benefit ratio an important issue for some patients. Moreover, steroids pulse therapy with a large dose may induce a transient worsening. Thus, other immunosuppressive agents have to be added.

Chinese practitioners of TCM frequently use herbal compounds of Chinese proprietary medicine together with active components of Western medicines [21]. Herbal medicines are believed by the general public to be safe, causing less side effects and less likely to cause dependency because they are “natural” [22]. In this study, we used Shenqi Fuzheng Injection and methylprednisolone pulse therapy together in the treatment group and the methylprednisolone pulse therapy only as a positive control in the control group so as to compare the effect of Shenqi Fuzheng Injection, with a positive result as demonstrated here. TCM holds that MG is primarily caused by spleen deficiency and qi weakness, and the treatment of this disease should be based on the principle of invigorating spleen and replenishing qi. In TCM theory, the spleen and stomach, which are responsible for the transformation and transportation of food, are the source of qi and blood production. When these two organs become deficient and cannot carry out their normal functions, there will be poor appetite, exercise intolerance, lethargy, general weakness, fatigue, weakness of the extremities, and a pale tongue. Because of qi deficiency, the clear yang cannot ascend, resulting in loose stools, diarrhea, and dysentery. In severe cases of spleen and stomach deficiencies, the yang qi may be unable to hold the organs in their normal position, causing prolapse of such internal organs as the stomach, uterus, and rectum. Moreover, since yang qi, unable to ascend, sinks into yin, it causes spontaneous sweating and a weak, deep pulse.

Shenqi Fuzheng compriss Radix Astragali (Huangqi) and Radix Codonopsis (Dangshen). Radix Codonopsis can supplement qi, promote the production of body fluid, and regulate the Spleen and Stomach. Moreover, Radix codonopsis can also promote the phagocytic activity of the body's macrophage system and the function of the cellular and fluid immune system, thus increasing the body's ability to counteract diseases. Radix Astragali can strengthen the spleen, replenish qi, increase the yang qi, and restore prolapse of organs. The combination of these two herbs can enhance the total effect by invigorating spleen and replenishing qi to support healthy energy. The effect of Shenqi Fuzheng Injection on the alleviation of the severity and duration of transient worsening of MG during steroids pulse therapy proved the correctness of TCM theory regarding the pathogenesis of MG.

Furthermore, from the perspective of modern sciences, these two components of Shenqi Fuzheng Injection do regulate the immune system of the body. The active components of Radix Astragali include polysaccharides, flavonoids and saponins, and the active components of Radix codonopsis include polysaccharides. All these components are beneficial for autoimmune disease because they can inhibit the production of serum autoantibodies and total IgG [23]. Moreover, Bu-Zhong-Yi-Qi-Tang, which contains the two components of Radix astragali and Radix codonopsis, has been proved to be able to strengthen antibody response in upper respiratory mucosal immune system in vivo by enhancing influenza virus-specific IgA and IgG antibody titers in nasal cavity and sera [24]. Bu-Zhong-Yi-Qi-Tang can also suppress nasal inflammation by an anti-inflammatory effect, thus being beneficial to patients with perennial allergic rhinitis [25]. 

This study has some limitations because it has only limited number of patients. A large number of patients are needed for future rigorous study to further prove the effect of Shenqi Fuzheng Injection in alleviating the severity and duration of transient worsening of MG during steroids pulse therapy.

In summary, this study indicates that Shenqi Fuzheng Injection can alleviate the severity and the duration of transient worsening of MG during steroids pulse therapy and has few side effects.

## Figures and Tables

**Table 1:** Demographic data (mean ± SD).

Groups	No.	Gender		Age (y)	Course (y)	MG classification				
		M	F			I	IIa	IIb	III	IV
Treatment	32	14	18	4–60 (37.0 ± 1.0)	0.5–17 (8.2 ± 0.5)	5	5	17	4	1
Control	34	17	18	6–58 (40.0 ± 0.5)	0.5–20 (8.4 ± 1.0)	6	5	18	3	2

No significant difference (*P* > 0.05) exists between the two groups.

**Table 2:** Clinical absolute and relative scores for patients with MG.

Ptosis (upper eyelid position)	Upper eyelid fatigue test (sec)	Eyeball horizontal movement (white of the eye, mm)	Upper limb fatigue test—arm outstretched (sec)	Lower limb fatigue test—knee and hip flexion at 90° (sec)	Facial muscles	Chewing and swallowing	Respiratory muscle
0 = normal	0 = >60	0 = ≤2, no diplopia	0 = >120	0 = >120	0 = normal with eyelashes all covered by eyelids	0 = normal function	0 = normal function
1 = 10–2 o'clock	1 = 31–60	1 = 3-4, with diplopia	1 = 61–120	1 = 61–120	1 = slightly reduced eye closure strength, eyelashes incompletely covered	2 = fatigue with solid food, prolonged food intake but normal food amount	2 = shortness of breath with movement or exertion
2 = 9–3 o'clock	2 = 16–30	2 = 5–8, with diplopia	2 = 31–60	2 = 31–60	2 = reduced eye closure strength, eyelashes all exposed outside	4 = fatigue with solid food, prolonged and decreased food intake	4 = shortness of breath with walking on a leveled area
3 = 8–4 o'clock	3 = 6–15	3 = 9–12, with diplopia	3 = 11–30	3 = 11–30	3 = can neither close eyelids nor puff up cheeks	6 = semifluid food only	6 = shortness of breath with sitting still
4 = 7–5 o'clock	4 = ≤5	4 = >12, with diplopia	4 = 0–10	4 = 0–10	4 = cannot pout with a masklike face	8 = nasal feeding only	8 = mechanical ventilation

Clinical relative score (CRS) = (pretreatment absolute score − posttreatment absolute score)/pretreatment absolute score.

Effect score: (1) complete remission if CRS ≥ 95%, (2) basic remission if CRS 80–95%, (3) marked improvement if CRS 50–80%, (4) improvement if CRS 25–50%, and (5) ineffectiveness if CRS < 25%.

**Table 3:** The clinical absolute and relative scores in patients with transient worsening.

Groups	No.	Absolute scores			Relative scores
		Pretreatment	Transient worsening	Posttreatment	
Treatment	29	10–52 (mean 30.5 ± 2.5)	16–64 (mean 47.3 ± 2.6)*	0–16 (mean 6.8 ± 0.8)	0.438–1 (mean 0.768 ± 0.026)
Control	29	10–54 (mean 25.6 ± 3.1)	28–60 (mean 50.5 ± 2.2)^∗#^	0–20 (mean 8.3 ± 1.2)	0.333–1 (mean 0.672 ± 0.036)

*indicates a significant difference (*P* < 0.000) compared with pretreatment.

^#^indicates a significant difference (*P* < 0.05) compared with the treatment group.

**Table 4:** Treatment effect at the end of the treatment course.

Effect results	Treatment group (no.)	Control group (no.)
Complete remission	4	2
Basic remission	11	6
Marked improvement	16	24
Improvement	1	2
Ineffectiveness	0	0

The number indicates the number of patients. More patients got remission or better improvement in the treatment group than in the control group.
